# Development and Validation of a Functional Antibody Assay for Evaluating Protein-Based Pneumococcal Vaccines

**DOI:** 10.3390/vaccines14020127

**Published:** 2026-01-27

**Authors:** Jiangjiao Li, Kang Li, Youyou Wang, Yang Huang, Xiuwen Sui, Xiao Xu, Huijing Du, Bochao Wei, Ying Yang, Jinming Zhang, Liang Kong, Tao Zhu, Bin Wang

**Affiliations:** 1 State Key Laboratory of Drug Regulatory Science, NHC Key Laboratory of Research on Quality and Standardization of Biotech Products, National Institutes for Food and Drug Control, Beijing 102629, China; lijiangjiao@nifdc.org.cn (J.L.);; 2 CanSino Biologics Research Center, Tianjin 300457, China

**Keywords:** *Streptococcus pneumoniae*, Pneumococcal surface protein A, pneumococcal vaccines, functional antibody, validation

## Abstract

Background: *Streptococcus pneumoniae* (Spn) is a leading bacterial pathogen responsible for severe invasive diseases, including meningitis, sepsis, and pneumonia. Current pneumococcal vaccines, which are all based on capsular polysaccharide antigens, provide limited protection and are further compromised by post-vaccination serotype replacement. Pneumococcal surface protein A (PspA), a highly conserved virulence factor expressed across diverse serotypes, has emerged as a promising candidate antigen for novel protein-based vaccines. However, progress in this field has been hindered by the absence of standardized in vitro functional antibody assays. Methods: This study established a robust functional antibody detection method for PspA-based protein vaccines by modifying the conventional multiplex opsonophagocytic killing assay (MOPA), originally designed for polysaccharide-based vaccines. Using polymerase chain reaction (PCR) and enzyme-linked immunosorbent assay (ELISA) typing, a target strain panel was selected and developed to include representative strains from PspA Family 1-Clade 2 and Family 2-Clades 3 and 4. The MOPA protocol was optimized by extending the phagocytic reaction time to enhance sensitivity. Specificity was confirmed through recombinant PspA competitive inhibition assays. Results: The assay demonstrated high linearity (R^2^ ≥ 0.98) between opsonophagocytic index (OI) and serum dilution, along with acceptable repeatability (CV ≤ 30%) and intermediate precision (CV ≤ 50%). Both preclinical and clinical serum samples exhibited potent bactericidal activity against diverse PspA families, independent of capsule type. Conclusions: This study provided a standardized framework to support the development and regulatory assessment of protein-based pneumococcal vaccines.

## 1. Introduction

*Streptococcus pneumoniae* (Spn) is a primary bacterial pathogen, causing serious human diseases, such as meningitis, sepsis, and pneumonia [1,2]. Capsular polysaccharides are key virulence factors in Spn, forming the basis of current vaccine development efforts [3,4]. Despite the identification of more than 100 pneumococcal serotypes, differentiated by diverse capsular polysaccharides (CP) [5,6,7], existing vaccines, including polysaccharide and polysaccharide-protein conjugate types, cover the most prevalent serotypes [3], such as conjugate vaccines PCV13 (Pfizer Inc., New York, NY, USA), PCV15 (Merck & Co., Rahway, NJ, USA), PCV20 (Pfizer Inc.), and polysaccharide vaccine PPSV23 (Merck & Co.). Challenges, including limited serotype coverage, serotype replacement after vaccination [8,9,10,11,12], and rising antibiotic resistance necessitate new vaccine strategies, such as pneumococcal protein vaccines, to address a wider range of serotypes [3,13,14,15,16,17].

Conserved pneumococcal proteins were investigated as potential vaccine components [18,19]. Pneumococcal surface protein A (PspA) is a promising antigen, playing a role in combating pneumococcal pneumonia, and it is widely present across most Spn serotypes [20,21,22]. Antibodies generated in response to recombinant PspA have demonstrated efficacy in animal models [23,24,25,26]. Structurally, PspA is categorized into three families and six clades based on sequence variation [27,28,29]. Epidemiological Chinese studies indicated that Spn strains primarily express PspA from Families 1 and 2, and Family 3 is notably absent [20]. The observed cross-reactivity of antibodies in the same PspA family indicates that a vaccine incorporating antigens from both Families 1 and 2 may potentially provide protection against a majority of Spn strains. The pneumococcal recombinant protein vaccine developed by companies, such as GSK [30] and Pasteur [31], has entered phases I and II clinical trials, including detoxified Ply derivative (PlyD1), pneumococcal histidine triad protein (PhtD), and pneumococcal choline-binding protein (PcpA). CanSino Biologics Inc. (Tianjin, China) has recently developed an innovative recombinant pneumococcal protein vaccine, involving three PspA antigens (P3296, P5668, and PRX1). PRX1 was derived from Clade 2 of PspA Family 1, while P3296 and P5668 belonged to Clades 3 and 4 of PspA Family 2, respectively. The vaccine also incorporated PlyLD, a modified variant of pneumolysin. This vaccine is currently in the phase I clinical trial stage. Despite the growing interest in developing PspA-based protein vaccines, evaluating their efficacy remains challenging due to the lack of reliable in vitro functional antibody assays.

The multiplexed opsonophagocytic killing assay (MOPA) mimics the in vivo immune opsonization of pathogens through in vitro testing, enabling direct measurement of functional antibodies capable of mediating phagocytic bactericidal activity in vaccine immune sera. This method demonstrates a strong correlation with vaccine protective efficacy [32,33] and has been successfully applied in evaluating the effectiveness of various multivalent pneumococcal polysaccharide vaccines, such as the 13-valent and 20-valent pneumococcal polysaccharide conjugate vaccines [34,35]. While several recombinant pneumococcal protein vaccines are currently undergoing clinical trials, MOPA has not been utilized to assess their antibody bactericidal activities. Traditional MOPA, which is more effective for evaluating anti-polysaccharide antibodies, is less appropriate for measuring anti-PspA antibodies. Establishing a reliable in vitro assay for detecting opsonic antibodies specific to PspA may significantly advance the development of PspA-based vaccines.

This study detailed the development and validation of an optimized MOPA designed to quantify protection-mediating antibodies induced by protein-based pneumococcal vaccines (PBPVs). Building upon the standard MOPA framework, critical modifications were introduced to enable accurate functional assessment of recombinant vaccines targeting PspA. The assay’s robust performance in both preclinical and clinical samples was further demonstrated, reflecting its reliability for evaluating PspA-specific antibody activities, providing a standardized platform for evaluating PspA vaccine immunogenicity.

## 2. Materials and Methods

### 2.1. Strains, Cells, and Complements

Spn strains, including TR6A, OP4, TR11A, ST14, and OP17F, were obtained from the University of Alabama at Birmingham (Birmingham, AL, USA). Strain F38 was gifted by Tsinghua University (Beijing, China). The HL60 cell line was procured from the American Type Culture Collection (ATCC; Manassas, VA, USA), and complements were obtained from both the China National Institutes for Food and Drug Control (Beijing, China) and CanSino Biologics Inc. All procedures involving live *Streptococcus pneumoniae* strains were conducted in a Biosafety Level 2 (BSL-2) laboratory.

### 2.2. Recombinant Proteins

The recombinant proteins P3296, P5668, and PRX1 were obtained from the PspA sequences of *Streptococcus pneumoniae* strains 3296, 5668, and RX1, respectively (sequences sourced from the National Center for Biotechnology Information: https://www.ncbi.nlm.nih.gov/, accessed on 5 July 2011). Specifically, strain 3296 is classified under PspA Family 2, Clade 3; strain 5668 under Family 2, Clade 4; and strain RX1 under Family 1, Clade 2. PlyLD, a leucine-to-aspartic acid point mutant at position 460 of the native Ply protein, was also utilized in this study. The recombinant PspA proteins (P3296, P5668, and PRX1) and the detoxified pneumolysin derivative PlyLD were designed as candidate vaccine antigens. They were expressed in *E. coli* and subsequently purified by CanSino Biologics Inc. for usage in this study.

### 2.3. Vaccines

The monovalent vaccine and the tetravalent recombinant pneumococcal protein vaccine (comprising P3296, P5668, PRX1, and PlyLD) were manufactured by CanSino Biologics Inc. [36].

### 2.4. Serum Samples

Rabbit serum samples, including polyclonal sera from animals immunized with either the monovalent or tetravalent vaccines, were prepared by CanSino Biologics Inc. These vaccines contained P3296, P5668, PRX1, and PlyLD. Human serum samples were collected from participants who aged 18–49 years participating in clinical phase Ia for the recombinant pneumococcal protein vaccine. The study protocol was approved by the Ethics Committee of Henan Provincial Center for Disease Control and Prevention (Zhengzhou, Henan, China; Approval No. 2019-YM-010-02). Written informed consent was obtained from all participants prior to their enrollment in the study.

### 2.5. PspA Typing of Spn Strains

#### 2.5.1. Polymerase Chain Reaction (PCR)

Typing of PspA families in Spn strains was determined using PCR as previously described, with some modifications [20,27,29]. In brief, genomic DNAs from each Spn strain were isolated, and PspA genes were amplified using specific oligonucleotide primers. For PspA Family 1, primers LSM12 (5’-CCGGATCCAGCGTCGCTATCTTAGGGGCTGGTT-3’) and SKH63 (5’-TTTCTGGCTCATYAACTGCTTTC-3’) were used, while for PspA Family 2, primers SKH52 (5’-TGGGGGTGGAGTTTCTTCTTCATCT-3’) along with LSM13 (5’-GCAAGCTTATGATATAGAAATTTGTAAC-3’) and SKH2 (5’-CCACATACCGTTTTCTTGTTTCCAGCC-3’) were employed. The PCR amplification was performed using the TransFast^®^ Taq DNA Polymerase commercial kit (TransGen Biotech, Beijing, China; catalog no. AP101-12) according to the manufacturer’s instructions. The thermal cycling protocol consisted of an initial denaturation at 95 °C for 3 min, followed by 30 cycles of 95 °C for 30 s, annealing at 58–62 °C for 30 s, and extension at 72 °C for 30 s. This was capped with a final extension at 72 °C for 10 min, and the reaction was held at 4 °C. Primer selection was based on the optimal annealing temperatures. The PCR products were then separated on a 1% agarose gel through gel electrophoresis and subjected to DNA sequencing for PspA protein type determination via sequence alignment. The classification of PspA families and clades was determined by aligning the obtained amino acid sequences of the α-helical region against a reference set of known PspA family/clade sequences from the literature [27]. The sequence similarity, expressed as the percentage of identity, was utilized to assign each strain to the PspA clade with the highest alignment match.

#### 2.5.2. Enzyme-Linked Immunosorbent Assay (ELISA)

The ELISA protocol was adapted from the literature [29], with modifications to determine the PspA subtype of each strain by assessing the reactivity of *Streptococcus pneumoniae* lysates with three rabbit anti-PspA antisera: PspA RX 1 (Family 1, Subclass 2), PspA 3296 (Family 2, Subclass 3), and PspA 5668 (Family 2, Subclass 4) (CanSino Biologics Inc.).

*Streptococcus pneumoniae* lysates were diluted 1:5 in 0.01 M PBS and added to a 96-well plate (Corning Inc., Corning, NY, USA), followed by overnight incubation at 4 °C. After incubation, the liquid was discarded, and plates were thrice washed, with each wash lasting 3 min using PBST washing buffer (Solarbio, Beijing, China). Blocking was performed with 2% bovine serum albumin (BSA; Solarbio, Beijing, China) for 1 h at room temperature. Subsequently, PspA antiserum (diluted 1:10,000) was added, and plates were incubated at 37 °C for 1 h. Two negative controls were included in parallel: (1) wells without lysate coating, while with antiserum addition, and (2) wells coated with lysate, while incubated with non-immune rabbit serum. After antiserum incubation, the plates were thrice washed with PBST. Freshly diluted horseradish peroxidase (HRP)-conjugated anti-rabbit IgG (1:200,000; ZSGB-BIO) was added, and the plates were incubated at 37 °C for 30 min, followed by three additional washes. Subsequently, 3,3’,5,5’-Tetramethylbenzidine (TMB) substrate (Sigma-Aldrich, St. Louis, MO, USA) was added to each well and incubated at 37 °C for 15 min for color development. Absorbance at 450 nm (A450) was measured using a microplate reader.

The reactivity of each *Streptococcus pneumoniae* lysate with the respective rabbit antiserum was expressed as the ratio of the sample A450 value to that of the negative control (wells containing non-immune rabbit serum). A ratio greater than 2 was considered positive.

Because most *S. pneumoniae* lysates reacted with two or all three of the PspA antisera, the final subtype assignment for each lysate was determined based on the antiserum that demonstrated the strongest reactivity.

### 2.6. Modified MOPA for PBPV Evaluation

The standard MOPA protocol, optimized for polysaccharide antibodies, underwent modifications for protein antigens due to potential differences in antibody-mediated opsonization kinetics. According to preliminary experiments that revealed suboptimal assay sensitivity, a systematic evaluation of key parameters was conducted, including effector cell concentration, bacterial inoculum size, and incubation conditions. Through this process, phagocytic reaction time was identified as the most critical factor requiring optimization. The standard MOPA protocol [37] involving 45-min incubation of opsonized bacteria, differentiated HL-60 cells, and complement with shaking at 700 rpm, was modified to assess the effects of different incubation time points. Conditions tested included shortened (30 min) and extended (75 min) durations, in addition to the conventional 45-min control. For the assay, serum samples were serially diluted threefold in opsonization buffer, mixed with target strains (representatives of PspA subtypes), and incubated at 700 rpm for 30 min at room temperature. Phagocytes and baby rabbit complement were added, and the reactions were incubated at 700 rpm, 37 °C, and 5% CO_2_ for each designated time period. Reactions were terminated by ice quenching, and the samples were plated on Todd-Hewitt Yeast Agar plates. Surviving colonies were enumerated after overnight incubation. Opsonic index (OI) values and the maximum bactericidal rate were calculated using Opsotiter software (Version 3) (WHO Pneumococcal Reference Laboratory, Birmingham, AL, USA). The OI value was defined as the reciprocal of the interpolated serum dilution required to achieve 50% bacterial killing, relative to control wells containing bacteria, complement, and phagocytic cells, without testing serum (serum-free control). A pooled serum sample from rabbits immunized with a tetravalent pneumococcal protein-based vaccine (produced by CanSino Biologics Inc.) was utilized as the quality control (QC) serum to ensure the reliability and consistency of the assay.

### 2.7. Verification of the Modified MOPA Method

#### 2.7.1. Specificity

Ten human serum samples were initially pre-absorbed with an equal ratio mixture of recombinant proteins P3296, P5668, PRX1, and PlyLD (1:1:1:1). These samples were thereafter exposed to the target pneumococcal strains, followed by the addition of complement and phagocytic cells to perform the opsonophagocytic assay. After incubation, the reaction mixtures were plated onto bacterial culture plates and incubated overnight. The resulting bacterial colonies were subsequently photographed, and the inhibition rates were determined by comparing the OI values before and after the adsorption process.

#### 2.7.2. Linearity

Three human serum samples were diluted at ratios of 1:9, 1:27, 1:81, 1:243, and 1:729 for MOPA. The linearity of the relationship between OI values and dilution ratios was evaluated by converting both the OI values and dilution ratios to base-3 logarithms and analyzing the correlation between the two.

#### 2.7.3. Repeatability

Six human serum samples were randomly selected, and each sample was analyzed in six replicates during a single day by a single operator. The degree of repeatability was quantified by calculating the coefficient of variation (CV) for these assays.

#### 2.7.4. Intermediate Precision

Nine human serum samples were randomly selected and analyzed by multiple operators on different days using a panel of six target bacterial strains. The intermediate precision of the method was determined by calculating the inter-assay coefficient of variation (CV) across these replicate measurements.

### 2.8. Application of the Modified Method

The validated MOPA method was then applied to analyze rabbit serum samples immunized with either the monovalent or tetravalent vaccines, as well as human serum samples from a Phase I clinical trial of the PBPV in adults (identifier: NCT04087460).

## 3. Results

### 3.1. PspA Classification of Pneumococcal Strains

#### 3.1.1. PCR-Based Typing

The primary region of the PspA protein, being crucial for subtype classification, consisted of five segments, including an α-helical region. Amino acid sequences from subtypes 1–5, corresponding to different strains, were referenced as described previously [27]. For each clade, a sequence that closely matched the literature reference was selected as the standard for making comparison.

**Figure 1:**
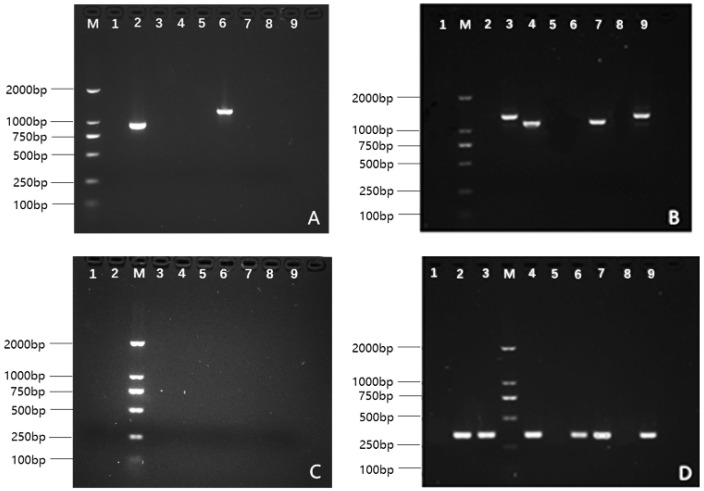
PCR-Based PspA Typing of Spn strains. Panel (**A**) (Family 1-specific PCR): Shows positive results in lanes 2 and 6, corresponding to strains TR6Aand F38, respectively. Panel (**B**) (Family 2-specific PCR): Displays positive results in lanes 3, 4, 7, and 9, for strains TR11A, ST14, OP17F, and OP4, respectively. Panel (**C**) (Family 3-specific PCR): Indicates no positive PCR results. Panel (**D**) (Spn-specific PCR): Reveals positive results in lanes 2, 3, 4, 6, 7, and 9, which match strains TR6A, TR11A, ST14, F38, OP17F, and OP4, respectively.

**Figure 2:**
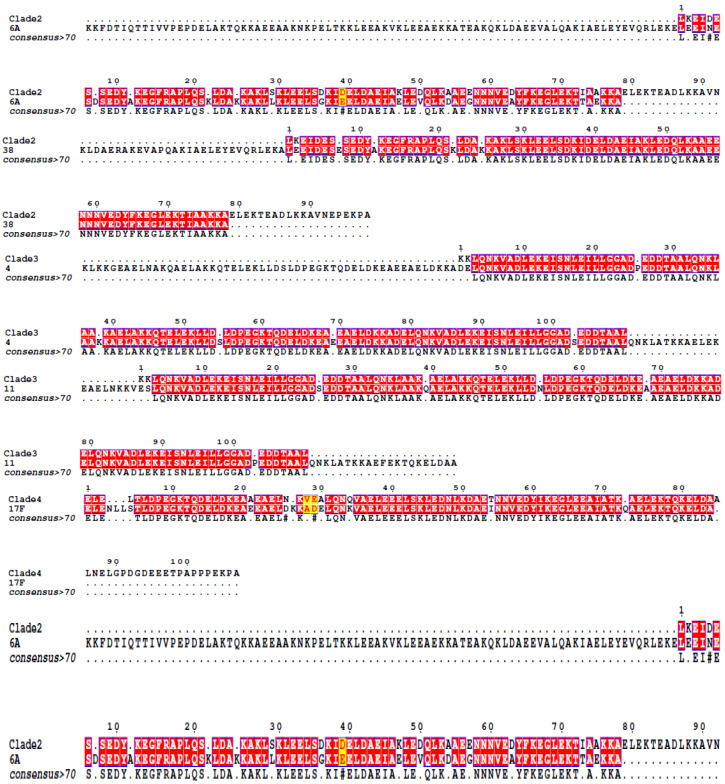
Sequence alignment of the α-helical region of PspA from study strains against reference sequences. The six study strains analyzed are: 6A (TR6A), 38 (F38), 4 (OP4), 11 (TR11A), 17F (OP17F), and 14 (ST14). Reference strains for each PspA clade were obtained from the literature [27]: Clade 2 (strain Rx1, GenBank accession no. M74122), Clade 3 (strain EF3296, GenBank accession no. AF071816), and Clade 4 (strain EF5668, GenBank accession no. U89711). Identical matches to the reference sequences are highlighted in red.

The amino acid sequences detected in this study were then aligned with these reference sequences. Gel electrophoresis results of the PCR products are presented in Figure 1, and the amino acid sequence alignment is illustrated in Figure 2. Sequences that exactly matched the reference literature sequences are highlighted in red. The typing results for the strains are summarized as follows: Strain TR6A, belonging to PspA Family 1, Clade 2, exhibited an 80% similarity to the reference sequence. Strain F38, also in Family 1, Clade 2, showed a 97% similarity. Strain OP4, from Family 2, Clade 3, demonstrated a 91.07% match to the reference. Strain TR11A, in Family 2, Clade 3, displayed a 95% similarity. Strain OP17F, classified under Family 2, Clade 4, exhibited an 89% similarity. Strain ST14, also in Family 2, Clade 4, showed a 97% similarity to its reference sequence.

#### 3.1.2. ELISA-Based Strain Typing

The ELISA-based strain typing was employed to classify various *Streptococcus pneumoniae* strains. This process led to the identification of strains TR6A and F38 as members of Family 1, Clade 2. In a similar vein, strains OP4 and TR11A were classified as Family 2, Clade 3. Additionally, strains OP17F and ST14 were categorized as part of Family 2, Clade 4. This methodical classification underlines the effectiveness of the ELISA method in the precise strain identification and confirms the findings from the PCR-based typing method.

### 3.2. Modification of the Standard MOPA Method

The standard MOPA method was optimized to improve its applicability for detecting bactericidal antibodies in pneumococcal protein vaccines. The first step in the optimization process involved selecting appropriate bacterial strains. Using PCR and ELISA screening techniques, six representative strains were identified from the MOPA bacterial panel. These strains were selected to involve three major PspA clades and were subsequently employed as target strains for further testing. Initially, variations in cell concentrations and bacterial loads were tested; however, these adjustments did not enhance the sensitivity of the assay in detecting PspA functional antibodies. A key advancement was made by systematically optimizing the incubation time for effector cells and complement, in which three durations were tested (30, 45, and 75 min). Significant differences were identified in the assay outcomes based on the incubation time. In particular, extending the incubation time resulted in a significant increase in the OI values for rabbit serum samples, and the maximum bactericidal rate was improved from 21% to 99%, as illustrated in Table 1. These results demonstrated that a longer incubation period for complement and effector cells could significantly enhance the sensitivity of the assay for detecting PspA functional antibodies. Importantly, the OI values for the negative control serum remained consistently negative across all incubation time points, demonstrating that the extended incubation period did not induce any false-positive results. Consequently, an incubation period of 75 min was determined to be the optimal duration for this method.

### 3.3. Validation of the Modified MOPA Method

The refined method’s reliability was systematically assessed using antisera from PspA-vaccinated individuals to ensure consistent measurement of functional antibodies. For the specificity validation, serum samples were initially pre-absorbed with recombinant proteins. The other validation parameters, including repeatability, intermediate precision, and linearity, were evaluated using the standard modified MOPA protocol without competitive inhibition.

#### 3.3.1. Specificity

To validate the specificity of the enhanced MOPA method, an antigen competitive inhibition approach was utilized. The process was summarized as follows: Ten human serum samples were initially pre-absorbed with an equal ratio mixture of recombinant proteins P3296, P5668, PRX1, and PlyLD (1:1:1:1). These samples were thereafter exposed to the target pneumococcal strains, followed by the addition of complement and phagocytic cells to perform the opsonophagocytic assay. After incubation, the reaction mixtures were plated on bacterial culture plates and left overnight. The resulting bacterial colonies were thereafter photographed, and the inhibition rates were quantitatively assessed using the OI values.

A significant proportion (80–100%) of the samples demonstrated ≥80% protein-specific competitive inhibition, confirming the specificity of the detection method (Table 2).

#### 3.3.2. Linearity

Linearity in an analytical method refers to its capacity to generate results that are directly proportional to the concentration of the analyte in a specified range. To assess the linearity of the MOPA in the present study, three human serum samples were randomly selected. The relationship between the OI values and the corresponding dilution factors for each serum sample was systematically analyzed. The findings of this analysis are detailed in Table 3.

**Figure 3:**
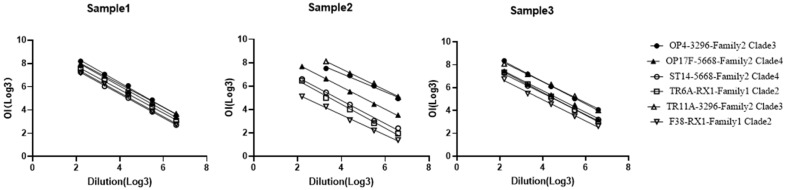
Linear Relationship Between Serum Dilution and OI Values. Three human serum samples were diluted at ratios of 1:9, 1:27, 1:81, 1:243, and 1:729 for MOPA. The linear relationship between each type of OI value and dilution ratio (both OI values and dilution ratios were converted to logarithms with base 3) was analyzed. ●, The strain of OP4 (Family 2 Clade 3); ▲, The strain of OP17F (Family 2 Clade 4); ○, The strain of ST14 (Family 2 Clade 4); □, The strain of TR6A (Family 1 Clade 2); △, The strain of TR11A (Family 2 Clade 3); ▽, The strain of F38 (Family 1 Clade 2). The differences in linear correlation were statistically significant (*p* < 0.05).

It was revealed that the linear slopes for the six target bacteria ranged between −0.7822 and −1.0594, and all R^2^ values exceeded 0.98. This indicates a strongly linear correlation, falling well within the acceptable range. The linear relationships for all three serum samples against the six target bacteria are visually presented in Figure 3, highlighting a consistently strong linear correlation, confirming the assay’s linearity across different serum samples and bacterial targets.

#### 3.3.3. Repeatability

Repeatability refers to the consistency of a given experiment’s results when it is repeated under the same conditions. To assess this for the MOPA, a single operator randomly selected six human serum samples and performed six parallel assays on each. The degree of repeatability was quantified by calculating the CV values for these assays. As indicated in Table 4, the CV values obtained for MOPA targeting the strains OP4, TR11A, OP17F, ST14, F38, and TR6A were all below the 30%, demonstrating a high level of consistency and reliability in the assay results.

#### 3.3.4. Intermediate Precision

Intermediate precision refers to the consistency of results when an assay is performed by different operators and at different times. For the MOPA, this was evaluated by randomly selecting nine human serum samples and having various operators conduct the assays at different time points using six target bacterial strains. The CV values were calculated to determine the MOPA method’s precision. As illustrated in Table 5, the results demonstrated a notable consistency across different operators and time points. With all CV values remaining below 50%, the assay demonstrated a high level of intermediate precision, indicating its reliability and robustness under varying conditions.

### 3.4. Application of the Modified MOPA Method

#### 3.4.1. Preclinical Applications in Vaccine Development

Rabbits were vaccinated with both monovalent vaccines (containing P3296, P5668, PRX1, and PlyLD, respectively) and a tetravalent vaccine, administered across three immunization doses at 14-day intervals. Serum samples were collected on days 0, 14, 28, and 42, corresponding to the pre-immune stage, first immunization, second immunization, and third immunization stages, respectively (Table 6). The modified MOPA was employed to evaluate the bactericidal activity of anti-PspA antibodies in the rabbit sera with six representative target strains.

As outlined in Table 6, the results showed that OI values significantly increased following immunization. For Family 1, subtype 2 strains TR6A and F38, the OI values in pre-immune serum were 24 and 53, respectively, which increased to 9474 and 48,306 following three doses of PRX1. In the case of Family 2, subtype 3 strains OP4 and TR11A, the OI values increased from 16 and 83 in pre-immune serum to 40,101 and 27,626, respectively, after immunization with P3296. For Family 2, subtype 4 strains ST14 and OP17F, there was a rise in OI values from 343 and 24 to 2041 and 2299, respectively, following the complete vaccination schedule.

According to the conventional MOPA, a fourfold increase in post-immune serum bactericidal activity compared with pre-immune levels is indicative of seroconversion. This study revealed 100% seroconversion following immunization with all three PspA antigens. Remarkably, bactericidal activity for P3296-specific antibodies increased by 2506-fold, while for P5668 and PRX1-specific antibodies, it rose by approximately 100-fold.

For the tetravalent vaccine, there was a substantial increase in serum bactericidal activity OI values from pre-immune levels of 17, 331, 13, 44, 196, and 23 to 1380, 18,558, 23,978, 19,653, 3838, and 1973 post-immunization. These results demonstrate a notable enhancement in functional antibody levels attributable to the tetravalent vaccine.

#### 3.4.2. Implementation in Human Clinical Serum Analysis

After confirming the reliability of the modified MOPA in measuring functional antibodies in rabbit immunization studies, the evaluation was extended to human vaccinee sera to assess clinical applicability. Healthy participants who aged 18–49 years were administered three doses of the tetravalent vaccine (CanSino Biologics Inc.), which included P3296, P5668, PRX1, and PlyLD antigens, at 30-day intervals. Serum samples were collected on days 0, 30, 60, 90, and 150, corresponding to the pre-immune stage, 30 days post-first immunization, 30 days post-second immunization, 30 days post-third immunization, and 60 days post-third immunization, respectively, as presented in Supplementary Table S1.

Thirteen randomly selected clinical trial participants and their longitudinally collected serum samples were analyzed using the modified MOPA. The results, as detailed in Supplementary Table S1, revealed that bactericidal activity significantly increased after vaccination.

For Family 1, subtype 2 strains TR6A and F38, 10 out of 13 participants demonstrated significantly enhanced bactericidal activity, ranging from 10- to 16,965-fold for TR6A, and 3- to 1963-fold for F38. In the case of Family 2, subtype 3 strains OP4 and TR11A, all 13 participants demonstrated significant increases in bactericidal activity, with ranges of 3 to 9254-fold for OP4, and 4 to 237-fold for TR11A. For Family 2, subtype 4 strains ST14 and OP17F, 12 out of 13 participants exhibited increases ranging from 3 to 6369-fold for ST14, while 10 out of 13 showed a 3 to 12-fold increase for OP17F. These results collectively demonstrated that the tetravalent vaccine could significantly enhance functional antibody levels against multiple pneumococcal strains from both Family 1 and Family 2.

## 4. Discussion

In contrast to pneumococcal polysaccharide vaccines, the protein-based vaccines provide broader coverage against a range of serotypes [38,39,40]. However, the clinical development of recombinant pneumococcal protein vaccines has been somewhat slow, partly due to the lack of appropriate methods for efficacy evaluation. The classical MOPA, designed to measure anti-capsular opsonic antibodies [41,42,43,44], fails to detect protein-specific functional responses due to distinct effector mechanisms. Earlier attempts to adapt MOPA for PspA vaccines [45] were constrained by narrow strain coverage and unvalidated clinical utility, necessitating responding to two critical questions: (1) whether the method can be generalized across phylogenetically distinct PspA families/subtypes, and (2) whether the method is appropriate for standardized clinical assessment of recombinant PspA vaccines.

In MOPA, the selection of target strain corresponds to the specific category of bacteria (e.g., serotype or protein type) that functional antibodies in serum can clear and eliminate [46,47]. Utilizing a broader range of target strains enhances the diversity of serotypes or protein types that can be identified as bactericidal. Previous studies, such as those conducted by Daniels et al. [45], utilized a single target strain, which limited the scope of detectable bactericidal categories. This study expanded the range of target strains, employing six bacterial strains representing PspA families 1 and 2. These strains cover over 99.5% of the pneumococcal strains prevalent in China [20], thereby validating their appropriateness for the efficacy evaluation of PBPV immunization.

The phagocytosis step is the critical phase of the MOPA, involving antibody-mediated bacterial clearance by phagocytes and complement [37,48]. The optimal co-incubation duration of the sample-bacteria-phagocyte-complement system is critical, as insufficient time leads to suboptimal opsonophagocytic activity, whereas prolonged incubation compromises assay practicality. For polysaccharide-based vaccines, a standard incubation time of 45 min is typically used. However, due to the unique characteristics of protein-based vaccines, MOPA typically exhibits lower sensitivity when applied to those vaccines. Through systematic optimization, this study revealed that 75-min incubation could significantly improve detection. It was confirmed that this extended incubation could enhance the bactericidal activity of protein vaccine-induced antibodies without causing false positives in pre-immune samples, making it appropriate for functional antibody assessment of protein-based vaccines. Following comprehensive validation, including specificity, linearity, repeatability, and intermediate precision, the modified MOPA method demonstrated robust detection of PspA-specific functional antibodies in human sera.

After validating the improved MOPA method, the bactericidal potential of rabbit serum samples was initially examined, which were immunized with both monovalent and tetravalent protein-based pneumococcal vaccines. Phase I clinical human serum samples from trials of the recombinant pneumococcal protein vaccine were subsequently selected for evaluation. The results obtained from rabbit serum samples demonstrated that immunization with antigens effectively induced functional antibodies, targeting specific families or subclasses of the PspA protein in *Streptococcus pneumoniae*. Notably, evidence of cross-protection was found; for instance, rabbit serum immunized with the PRX1 protein generated functional antibodies against both pneumococcal family 1, subclass 2, and family 2, subclass 4. This cross-protection among different PspA families and subclasses is particularly significant, given the extensive sequence variability of PspA across pneumococcal strains. A vaccine capable of inducing antibodies that recognize multiple PspA variants would address the limitations of strain-specific immunity, enhance protection against both circulating and emerging pneumococcal serotypes, and mitigate the risk of vaccine escape.

The bactericidal results from human serum samples revealed high antibody levels 30 days post-first immunization, with a slight reduction in antibody levels after the second and third immunizations. This decline may be attributed to the presence of pre-existing immunity against *Streptococcus pneumoniae* in adults, where the initial dose functioned as a booster, reaching a specific antibody threshold. A subset of vaccinees exhibited a relatively lower OI value against the TR6A strain (Family 1, Clade 2) compared with other strains. Several factors may contribute to this variability, including differences in the pre-existing immune repertoire or potential subtle differences in PspA expression or its accessibility on the TR6A strain’s surface. Despite this, the assay successfully detected strong responses against the other Family 1, Clade 2 strain (F38), as well as strains from other clades, confirming the vaccine’s ability to generate broad functional antibodies. The optimized MOPA method demonstrated its capability to detect functional antibodies against various PspA family strains in human serum, highlighting the potential for broad, serotype-independent protection through conserved epitopes across multiple protein subclasses.

## 5. Conclusions

In summary, this study developed a functional antibody detection method for protein-based pneumococcal vaccines by expanding the target strain repertoire to include a broader range of PspA Family 1 and 2 variants and refining the MOPA protocol. Through reliable methodological validation and successful application in both rabbit and human serum samples, it was revealed that this assay could reliably quantify bactericidal antibodies against the PspA protein. This research addressed the critical gap in validated functional assays for pneumococcal protein vaccines, a challenge that has previously hindered precise immunogenicity evaluation. The findings may provide a standardized framework for the development and regulatory assessment of protein-based pneumococcal vaccines.

## Figures and Tables

**Table 1:** Influence of Incubation Time on Opsonic Index (OI) Values.

PspA Family and Clades	Spn Serotype Strains	Incubation Time	OI Values		Maximum Bactericidal Rate	
			Rabit Serum	Negative Control	Rabit Serum	Negative Control
Family 1 and Clade 2	TR6A	30 min	2	2	24%	5%
		45 min	552	2	95%	6%
		75 min	1380	2	98%	3%
	F38	30 min	2	2	41%	19%
		45 min	2892	2	100%	15%
		75 min	6216	2	98%	15%
Family 2 and Clade 3	OP4	30 min	2	2	23%	5%
		45 min	4335	2	60%	3%
		75 min	16,458	2	95%	2%
	TR11A	30 min	227	2	80%	13%
		45 min	2190	2	97%	29%
		75 min	16,178	2	98%	26%
Family 2 and Clade 4	ST14	30 min	114	2	66%	44%
		45 min	1398	2	97%	23%
		75 min	5073	2	99%	21%
	OP17F	30 min	2	2	21%	36%
		45 min	1074	2	92%	42%
		75 min	6055	2	96%	7%

**Table 2:** Inhibition Rates for Human Serum 30 Days Post-Single Immunization.

Samples	Tested Spn Serotype Strains					
	F38	TR6A	OP4	TR11A	ST14	OP17F
1	99.95%	99.83%	99.86%	98.18%	93.30%	81.90%
2	99.94%	99.95%	87.64%	99.97%	89.73%	93.10%
3	40.42%	99.76%	99.98%	99.09%	61.37%	83.41%
4	77.71%	93.90%	90.84%	94.82%	89.32%	89.67%
5	99.95%	99.82%	98.65%	92.50%	76.00%	99.98%
6	99.91%	99.72%	86.49%	98.25%	94.36%	92.64%
7	99.98%	99.98%	99.99%	99.99%	99.99%	99.99%
8	89.00%	99.98%	94.15%	93.47%	97.13%	95.04%
9	83.64%	93.17%	97.37%	94.00%	97.91%	92.98%
10	99.52%	99.97%	99.98%	99.07%	99.96%	89.40%
Proportion	80.00%	100.00%	100.00%	100.00%	80.00%	100.00%

1. Inhibition rate (%) = [1 − (OI of sample with inhibitor/OI of sample without inhibitor)] × 100%. The OI values of uninhibited samples ranged from approximately 722 to 58163 across various serum samples and target strains. In contrast, the corresponding OI values in inhibited samples fell within a range of 2 to 4360. 2. Proportion (%) = (Number of samples with inhibition rate greater than 80%/Total number of samples) × 100%. 3. The final dilution of the human serum samples following absorption treatment was 1:10, which was performed prior to subsequent serial dilution in the MOPA.

**Table 3:** Linear Relationship between Opsonic Index (OI) Values and Dilution Ratios for Human Serum Samples.

Samples	PspA Family and Clades	Spn Serotype Strains	Linear Equation	R^2^
Sample 1	PRX1	TR6A	y = −1.0111x + 8.9251	0.9981
	(Family 1,Clade 2)	F38	y = −1.0274x + 8.7336	0.9931
	P3296	OP4	y = −1.0594x + 9.6453	0.9964
	(Family 2,Clade 3)	TR11A	y = −0.9909x + 9.2732	0.9996
	P5668	ST14	y = −1.0194x + 8.6122	0.9994
	(Family 2,Clade 4)	OP17F	y = −1.0421x + 9.3108	0.9962
Sample 2	PRX1	TR6A	y = −1.0258x + 7.7832	0.9926
	(Family 1,Clade 2)	F38	y = −0.8521x + 6.3437	0.9959
	P3296	OP4	y = −0.7822x + 9.2911	0.9857
	(Family 2,Clade 3)	TR11A	y = −0.908x + 10.116	0.9968
	P5668	ST14	y = −0.9862x + 7.9450	0.9916
	(Family 2,Clade 4)	OP17F	y = −0.9550x + 8.8879	0.9989
Sample 3	PRX1	TR6A	y = −0.9904x + 8.6926	0.9990
	(Family 1,Clade 2)	F38	y = −0.9274x + 7.8863	0.9955
	P3296	OP4	y = −0.9758x + 9.5033	0.9988
	(Family 2,Clade 3)	TR11A	y = −0.9179x + 9.2832	0.9947
	P5668	ST14	y = −0.9373x + 8.4320	0.9966
	(Family 2,Clade 4)	OP17F	y = −0.9454x + 8.6673	0.9947

**Table 4:** Repeatability of Opsonization Index (OI) Values in Human Serum Samples.

Samples	Family 1 Clade 2					
	TR6A			F38		
	GMT	SD	CV	GMT	SD	CV
1	4266	348	8%	7854	884	11%
2	4909	243	5%	7540	1069	14%
3	4555	909	20%	10,375	1730	16%
4	18,631	2495	13%	16,074	4415	26%
5	11,108	1044	9%	25,686	4422	17%
6	2413	565	23%	12,647	2001	16%
Samples	Family 2 Clade 3					
	OP4			TR11A		
	GMT	SD	CV	GMT	SD	CV
1	4642	136	3%	2999	261	9%
2	8851	1835	20%	13,035	1708	13%
3	27,463	3677	13%	17,603	2427	14%
4	11,783	1351	11%	13,265	2367	18%
5	38,050	5547	14%	50,169	4374	9%
6	42,070	5145	12%	50,606	8780	17%
Samples	Family 2 Clade 4					
	ST14			OP17F		
	GMT	SD	CV	GMT	SD	CV
1	4721	822	17%	6754	456	7%
2	2862	250	9%	4000	703	17%
3	6494	882	13%	8992	1356	15%
4	6689	929	14%	16,029	3701	23%
5	18,527	2859	15%	23,766	5090	21%
6	17,466	2001	11%	38,853	5376	14%

**Table 5:** Intermediate Precision for Opsonic Index (OI) Values of Human Serum Samples.

Samples	Family 1 Clade 2					
	TR6A			F38		
	GMT	SD	CV	GMT	SD	CV
1	2800	920	32%	3286	922	27%
2	580	166	28%	3430	996	28%
3	1009	392	36%	4311	1136	26%
4	1817	401	22%	4268	1525	34%
5	3405	957	27%	5660	1845	31%
6	3550	950	26%	12,042	3275	26%
7	16,919	3648	21%	20,445	6338	30%
8	9035	1975	21%	13,991	2954	21%
9	1874	423	22%	7116	1843	25%
Samples	Family 2 Clade 3					
	OP4			TR11A		
	GMT	SD	CV	GMT	SD	CV
1	19,473	6661	33%	36,591	4689	13%
2	23,349	8335	34%	35,174	11,987	33%
3	5389	1540	28%	3034	682	22%
4	9051	2390	26%	11,865	1539	13%
5	33,798	6368	19%	22,114	4557	20%
6	14,639	4477	30%	19,458	4424	22%
7	32,961	12,003	34%	42,365	7646	18%
8	41,185	8529	20%	40,576	3267	8%
9	44,251	8424	19%	45,338	6688	15%
Samples	Family 2 Clade 4					
	ST14			OP17F		
	GMT	SD	CV	GMT	SD	CV
1	5289	1011	19%	10,520	3346	31%
2	5058	1021	20%	11,032	2693	24%
3	5048	465	9%	8311	3354	38%
4	2719	634	23%	3717	816	21%
5	6513	1582	24%	9641	2195	22%
6	5659	1366	24%	15,308	3775	24%
7	12,495	2532	20%	27,905	10,636	36%
8	17,503	5745	32%	17,678	6409	35%
9	16,616	7982	45%	34,981	6112	17%

**Table 6:** Opsonic Index (OI) Values of Rabbit Serum at Various Immunization Stages.

Vaccines & Time Points		Family 1 Clade 2		Family 2 Clade 3		Family 2 Clade 4	
		TR6A	F38	OP4	TR11A	ST14	OP17F
PRX1 single antigen vaccination	Pre-VAC	24	53	18	174	272	23
	VACI	55	749	20	263	250	2
	VACII	2057	12,012	128	216	304	107
	VACIII	9474	48,306	132	177	532	113
P3296 single antigen vaccination	Pre-VAC	15	92	16	83	186	24
	VACI	146	49	5632	10,242	394	43
	VACII	124	65	35,070	34,857	792	109
	VACIII	55	47	40,101	27,626	689	107
P5668 single antigen vaccination	Pre-VAC	33	83	17	97	343	24
	VACI	252	127	466	1356	694	141
	VACII	222	1550	1695	1891	5170	1041
	VACIII	137	2082	1738	1912	2041	2299
4-Valent PBPV	Pre-VAC	17	331	13	44	196	23
	VACI	163	69,130	5422	6558	786	274
	VACII	1015	14,226	37,210	34,545	5378	2387
	VACIII	1380	18,558	23,978	19,653	3838	1973
Negative Control	Pre-VAC	32	248	13	98	196	23
	VACI	28	222	8	125	190	18
	VACII	33	228	12	148	182	25
	VACIII	35	253	17	176	222	26

Pre-VAC: Pre vaccination; VACI: After the first immunization; VACII: After the second immunization; VACIII: After the third immunization.

## Data Availability

The datasets of this study are available from the corresponding author upon reasonable request.

## Supplementary Materials

The following supporting information can be downloaded at: https://www.mdpi.com/article/10.3390/vaccines14020127/s1. Table S1. Opsonic Index (OI) Values of human Serum at Various Immunization Stages.
