# Supplementary material for: Development and Validation of a Functional Antibody Assay for Evaluating Protein-Based Pneumococcal Vaccines
Source: Vaccines (Basel). 2026 Jan 27;14(2):127. doi: 10.3390/vaccines14020127 (PMC12945081; doi:10.3390/vaccines14020127)
Supplement: Supplementary file 1 [file vaccines-14-00127-s001.zip › vaccines-4077558-supplementary.pdf]

Supplementary Table S1. Opsonic Index (OI) Values of human Serum at Various Immunization Stages.

Participants & Time points		Family 1 Clade 2		Family 2 Clade 3		Family 2 Clade 4	
		TR6A	F38	OP4	TR11A	ST14	OP17F
Participant 1	Pre-VAC	2	11	3386	3629	1240	1440
	30 Day post 1st VAC	454	1876	10,771	19,576	4167	8848
	30 Day post 2nd VAC	2	4274	9795	17,942	4168	7348
	30 Day post 3rd VAC	2	4712	11,389	16,663	3849	7166
	60 Day post 3rd VAC	2	2695	9960	10,333	3031	6655
Participant 2	Pre-VAC	2	260	533	1204	1112	2452
	30 Day post 1st VAC	947	5433	75,617	112,434	21,089	16,473
	30 Day post 2nd VAC	2018	1272	29,400	35,905	11,754	5157
	30 Day post 3rd VAC	2	984	14,440	23,241	6379	2710
	60 Day post 3rd VAC	2	383	14,380	16,575	3791	2727
Participant 3	Pre-VAC	442	2	3682	851	1314	717
	30 Day post 1st VAC	4476	3927	25,241	21,426	13,328	3948
	30 Day post 2nd VAC	2795	5334	12,539	14,899	8997	3032
	30 Day post 3rd VAC	3608	4870	9974	8901	5594	2589
	60 Day post 3rd VAC	2883	3002	6617	11,458	3563	1882
Participant 4	Pre-VAC	2	1028	388	751	898	6428
	30 Day post 1st VAC	5692	11,244	4096	4396	9432	14,043
	30 Day post 2nd VAC	4883	4012	3938	1707	5473	10,985
	30 Day post 3rd VAC	2359	4015	6467	3690	3946	10,334
	60 Day post 3rd VAC	2900	4044	6639	10,010	4389	6478
Participant 5	Pre-VAC	2	1798	1041	1009	1027	2634
	30 Day post 1st VAC	13,644	7342	28,472	15,504	16,652	14,675
	30 Day post 2nd VAC	2313	3201	5645	3299	7151	6890
	30 Day post 3rd VAC	1215	4053	5456	2965	3186	4882
	60 Day post 3rd VAC	2	3073	5185	2274	2444	3731
Participant 6	Pre-VAC	22	665	1502	3439	888	4091
	30 Day post 1st VAC	2	2109	24,931	13,173	1342	10,411
	30 Day post 2nd VAC	2	1424	19,857	10,091	1048	3121
	30 Day post 3rd VAC	2	2	12,270	10,225	1335	4160
	60 Day post 3rd VAC	2	2	16231	9167	778	2728
Participant 7	Pre-VAC	2	1058	31	3620	864	2733
	30 Day post 1st VAC	33,930	2198	32,428	21,470	5153	19,391
	30 Day post 2nd VAC	11,220	2352	19,221	18,528	2806	13,196
	30 Day post 3rd VAC	12,279	1864	11,531	16,123	3794	11,294
	60 Day post 3rd VAC	5268	1444	10,522	9937	2927	11,645
Participant 8	Pre-VAC	1396	13,077	2599	2663	4990	5148
	30 Day post 1st VAC	13,939	16,781	18,149	14,675	21,005	12,261
	30 Day post 2nd VAC	5314	7863	8129	8085	19,970	12,388
	30 Day post 3rd VAC	1930	5167	4366	2716	14,855	9794
	60 Day post 3rd VAC	1250	4231	3713	1594	12,125	7826
Participant 9	Pre-VAC	2	2	2773	125	515	1314
	30 Day post 1st VAC	1281	1459	36,028	29,571	7672	5856
	30 Day post 2nd VAC	2	2	11,789	4667	2176	3924

	30 Day post 3rd VAC	298	2	6163	3126	1896	2925
	60 Day post 3rd VAC	2	2	5527	3189	1625	3253
Participant 10	Pre-VAC	2	2084	1604	1416	819	9208
	30 Day post 1st VAC	2	1841	26,609	42,638	3963	9945
	30 Day post 2nd VAC	2	2670	17,691	16,882	5842	10,328
	30 Day post 3rd VAC	2	2028	16,928	20,667	4285	11,714
	60 Day post 3rd VAC	2	1698	18,032	18,998	5228	9924
Participant 11	Pre-VAC	2	4457	2877	3920	812	2986
	30 Day post 1st VAC	11,838	17,779	71,740	46,717	35,319	25,945
	30 Day post 2nd VAC	5957	10,584	37,787	27,647	14,984	17,054
	30 Day post 3rd VAC	4754	12,052	23,167	15,832	12,109	9346
	60 Day post 3rd VAC	4922	7079	21,356	11,439	17,282	13,639
Participant 12	Pre-VAC	2	393	798	949	133	885
	30 Day post 1st VAC	3467	6847	15,990	20,854	9213	10,284
	30 Day post 2nd VAC	1767	4870	21,165	30,386	5467	5150
	30 Day post 3rd VAC	2	3262	13,304	22,375	4748	5055
	60 Day post 3rd VAC	640	1927	9720	13353	2838	4438
Participant 13	Pre-VAC	2	275	2	974	2	2228
	30 Day post 1st VAC	11,200	12,726	18,508	52,420	12,738	9908
	30 Day post 2nd VAC	5673	17,655	20,903	43,155	35,220	9677
	30 Day post 3rd VAC	5552	8339	11,195	33,281	4931	8123
	60 Day post 3rd VAC	7942	6889	11,473	30,408	21,826	5810
